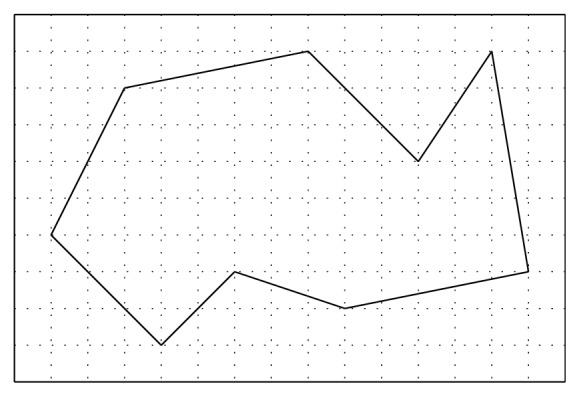# Pick's Theorem in Two-Dimensional Subspace of ℝ^3^

**DOI:** 10.1155/2015/535469

**Published:** 2015-02-23

**Authors:** Lin Si

**Affiliations:** College of Science, Beijing Forestry University, Beijing 100083, China

## Abstract

In the Euclidean space ℝ^3^, denote the set of all points with integer coordinate by ℤ^3^. For any two-dimensional simple lattice polygon *P*, we establish the following analogy version of Pick's Theorem, *k*(*I*(*P*) + (1/2)*B*(*P*) − 1), where *B*(*P*) is the number of lattice points on the boundary of *P* in ℤ^3^, *I*(*P*) is the number of lattice points in the interior of *P* in ℤ^3^, and *k* is a constant only related to the two-dimensional subspace including *P*.

## 1. Introduction

In the Euclidean plane ℝ^2^, a lattice point is one whose coordinates are both integers. A lattice polygon is a polygon with all vertices on integer coordinates. The area *A*(*P*) of a simple lattice polygon *P* can be given by celebrated Pick's theorem [1]

(1) $$\begin{array}{c} A\left(P\right)=I\left(P\right)+\frac{1}{2}B\left(P\right)-1, \end{array}$$

where *B*(*P*) is the number of lattice points on the boundary of *P* and *I*(*P*) is the number of lattice points in the interior of *P*.

Pick's formula can be used to compute the area of a lattice polygon conveniently.

For example, in Figure 1, *I*(*P*) = 60, *B*(*P*) = 15. Then, the area of the polygon is *A*(*P*) = 60 + 15 − 1 = 74.

There are many papers concerning Pick's theorem and its generalizations [2–5], which mostly be discussed in two dimensions.

Unfortunately, Pick theorem is failed in three dimensions. In 1957, John Reeve found a class of tetrahedra, named as Reeve tetrahedra later, whose vertices are

(2) $$\begin{array}{c} {\left(0,0,0\right)}^{T},{\left(1,0,0\right)}^{T},{\left(0,1,0\right)}^{T},{\left(1,1,r\right)}^{T}, \end{array}$$

where *r* is a positive integer.

All Reeve tetrahedra contain the same number of lattice points, but their volumes are different.

In this note, we discussed Pick's theorem in two-dimensional subspace of ℝ^3^. For any (*a*, *b*, *c*)^*T*^ ∈ ℤ^3^ with (*a*, *b*, *c*) = 1, that is, the greatest common factor of *a*, *b*, *c* is one, denote by *K*, *ax* + *by* + *cz* = 0, the two-dimensional subspace of ℝ^3^. Then we established the following theorem.

**Theorem 1 .**

If *P* is simple lattice polygon in the *K*, then the area of *P* is

(3) $$\begin{array}{c} k\left(I\left(P\right)+\frac{1}{2}B\left(P\right)-1\right), \end{array}$$

where *B*(*P*) is the number of lattice points on the boundary of *P* in ℤ^3^, *I*(*P*) is the number of lattice points in the interior of *P* in ℤ^3^, and *k* is the constant $(a^{3}+ab^{2})\sqrt{a^{2}+b^{2}+c^{2}}$.

**Remark 2 .**

Although the simple lattice polygon *P* is in the two-dimensional subspace *K*, the lattice points in *P* belong to ℤ^3^.

Let (*a*, *b*, *c*)^*T*^ = (1,0, 0)^*T*^ in the Theorem; then we can get Pick's theorem in some coordinate plane of ℝ^3^.

**Corollary 3 .**

If *P* is simple lattice polygon in the *K*, whose normal vector is (1,0, 0)^*T*^, then the area of *P* is

(4) $$\begin{array}{c} I\left(P\right)+\frac{1}{2}B\left(P\right)-1. \end{array}$$

## 2. Proof of Main Result

For any (*a*, *b*, *c*)^*T*^ ∈ ℤ^3^ with (*a*, *b*, *c*) = 1, there is two-dimensional subspace of ℝ^3^

(5) $$\begin{array}{c} ax+by+cz=0, \end{array}$$

whose normal vector is just (*a*, *b*, *c*)^*T*^. We denote this two-dimensional subspace by *K*.

By the theory of linear equations system, (−*b*, *a*, 0)^*T*^ and (−*c*, 0, *a*)^*T*^ are two linearly independent solutions of (5). We denote (−*b*, *a*, 0)^*T*^ by *α* and (−*c*, 0, *a*)^*T*^ by *β*. Obviously, *α* and *β* are also the basis of *K*.

**Lemma 4 .**

For any (*a*, *b*, *c*)^*T*^ ∈ ℤ^3^ with (*a*, *b*, *c*) = 1, there exists the lattice basis with the minimal area in the two-dimensional subspace *K*.

**Proof**

The area of parallelogram generated by *α* and *β* is

(6) 1a2+b2+c2a−b−cba0c0a =a3+ab2+ac2a2+b2+c2=aa2+b2+c2.

Denote (*a*, *b*, *c*)^*T*^ by *n*. For any lattice basis in *K*, *k*_1_ *α* + *k*_2_ *β* and *l*_1_ *α* + *l*_2_ *β*, where *k*_*i*_, *l*_*i*_ ∈ ℤ (*i* = 1,2) and $\left|\begin{array}{cc} k_{1} & l_{1}\\ k_{2} & l_{2} \end{array}\right|=0$. The area of parallelogram generated by *k*_1_ *α* + *k*_2_ *β* and *l*_1_ *α* + *l*_2_ *β* is

(7) 1a2+b2+c2n⋮k1α+k2β⋮l1α+l2β =1a2+b2+c2(n,α,β)×1000k1l10k2l2,

where $\left|\begin{array}{ccccc} n & \vdots & k_{1}\alpha +k_{2}\beta & \vdots & l_{1}\alpha +l_{2}\beta \end{array}\right|$ denote the determinant of *n*, *k*_1_ *α* + *k*_2_ *β*, and *l*_1_ *α* + *l*_2_ *β*.

Thus the lattice basis *k*_1_ *α* + *k*_2_ *β* and *l*_1_ *α* + *l*_2_ *β* have the minimal area if and only if $\left|\begin{array}{cc} k_{1} & l_{1}\\ k_{2} & l_{2} \end{array}\right|=1$.

Let *k*_1_ = 1, *k*_2_ = 0, *l*_1_ = 0, and  *l*_2_ = 1, and *α*, *β* are the lattice basis with the minimal area in the two-dimensional subspace *K*.

**Lemma 5 .**

For any (*a*, *b*, *c*)^*T*^ ∈ ℤ^3^ with (*a*, *b*, *c*) = 1, there exists the orthogonal lattice basis in the two-dimensional subspace *K*.

**Proof**

By Lemma 4, *α*, *β* are the lattice basis with the minimal area in the two-dimensional subspace *K*. By Schmidt orthogonalization, let

(8) γ1=α=−b,a,0T,γ2=β−(β,γ1)(γ1,γ1)=−c,0,aT−bca2+b2γ1=−c,0,aT−−b2ca2+b2,abca2+b2,0T=−a2ca2+b2,−abca2+b2,a3+ab2a2+b2T,

where (*β*, *γ*_1_) denote the usual inner product of *β*, *γ*_1_ in ℝ^3^.

Thus

(9) η1=γ1=α=−b,a,0T,η2=(a2+b2)γ2=−a2c,−abc,a3+ab2T

are the orthogonal lattice basis in the two-dimensional subspace *K*.

**Proof of Theorem.**

By Lemma 5, *η*_1_, *η*_2_ are the orthogonal lattice basis in the two-dimensional subspace *K*.

The area of parallelogram generated by *η*_1_, *η*_2_ is

(10) 1a2+b2+c2a−b−a2cba−abcc0a3+ab2 =a5+a3b2+ab2c2+a3c2+a3b2+ab4a2+b2+c2 =a(a4+2a2b2+b4+(a2+b2)c2)a2+b2+c2 =a(a2+b2)(a2+b2+c2)a2+b2+c2 =(a3+ab2)a2+b2+c2,

which just is the constant *k* in the theorem.

## Figures and Tables

**Figure 1:**